# Head mounted DMD based projection system for natural and prosthetic visual stimulation in freely moving rats

**DOI:** 10.1038/srep34873

**Published:** 2016-10-12

**Authors:** Tamar Arens-Arad, Nairouz Farah, Shai Ben-Yaish, Alex Zlotnik, Zeev Zalevsky, Yossi Mandel

**Affiliations:** 1 Faculty of Life Sciences, Optometry Track, Bar Ilan University, Ramat Gan, Israel; 2 Bar Ilan’s Institute for Nanotechnology and Advanced Materials (BINA), Bar Ilan University, Ramat Gan, Israel.; 3 Faculty of Engineering, Bar Ilan University, Ramat Gan, Israel

## Abstract

Novel technologies are constantly under development for vision restoration in blind patients. Many of these emerging technologies are based on the projection of high intensity light patterns at specific wavelengths, raising the need for the development of specialized projection systems. Here we present and characterize a novel projection system that meets the requirements for artificial retinal stimulation in rats and enables the recording of cortical responses. The system is based on a customized miniature Digital Mirror Device (DMD) for pattern projection, in both visible (525 nm) and NIR (915 nm) wavelengths, and a lens periscope for relaying the pattern directly onto the animal’s retina. Thorough system characterization and the investigation of the effect of various parameters on obtained image quality were performed using ZEMAX. Simulation results revealed that images with an MTF higher than 0.8 were obtained with little effect of the vertex distance. Increased image quality was obtained at an optimal pupil diameter and smaller field of view. Visual cortex activity data was recorded simultaneously with pattern projection, further highlighting the importance of the system for prosthetic vision studies. This novel head mounted projection system may prove to be a vital tool in studying natural and artificial vision in behaving animals.

Outer retinal degenerative diseases, are characterized by loss of photoreceptors, while inner retinal layers are relatively preserved^1^,^2^,^3^. The most common form of outer retinal degeneration diseases is Age-related Macular Degeneration (AMD) which is the major cause of vision loss in people over 65 in the Western world^4^, with no cure currently available. Another form of degenerative disease is Retinitis Pigmentosa (RP) which is the leading cause of inherited blindness in young adults, also with no current effective treatment available. Vision restoration in patients suffering from outer retinal degeneration can be obtained by bypassing the degenerated cells and the stimulation of the surviving inner retinal cells by various techniques; such as electrical stimulation^5^, ultrasound^6^, photoswitches^7^, or by optogenetics based treatment^8^,^9^. In all these methods, functional vision restoration methods rely on the ability to generate activation patterns with high spatial resolution. Several of these approaches (e.g. photovoltaic implants, optogenetics and photoswitch) rely on the projection of high intensity, localized light patterns at specific wavelengths onto the retina to achieve the desired patterned activation of retinal neurons.

In parallel to advances in vision restoration technologies there is a growing need for devising and developing advanced methods for assessing the spatial and temporal resolution of the obtained artificial vision in experimental animal models. Of main importance is the ability to project light stimuli in awake animals and record the induced visual cortical activity in response to the stimuli. The stimulation projection system should project patterned visual stimuli in the desired wavelength, at high light intensities and high spatial resolution, while covering a relatively large area of the retina. For example, when studying artificial visual performance in sighted animals using a photovoltaic array implant, it is crucial to activate the prosthesis with non visible light (at near IR wavelengths, 915 nm)^5^,^10^,^11^ while avoiding stimulation of the healthy retina by visible light. On the other hand, when studying optogenetics treated animals; activation of the retina is achieved by using blue light (peak activation of wavelength around 470 nm)^12^. Another important desired feature of the system is the ability to achieve a large field of view which will induce percepts and behavioral responses by the animal. This leads to the need for the stimulation of a relatively large retinal area with patterned light. Furthermore, in addition to the ability to project high spatial resolution and high power patterns at the desired light wavelengths, it is important to record animal behavior or electrophysiological response to various visual stimuli.

As part of the efforts towards the development of a system which fulfils all the characteristics previously mentioned, we present here the development and optical characterization of a novel head mounted, customized Digital Mirror Device (DMD) based projection system designed for experiments in behaving rats. The novel head mounted projection system is developed around a commercially available DMD projection system, which enables the projection of flexible light patterns with high spatial resolution. In our system (Fig. 1), the originally supplied Red LEDs is replaced and the DMD is illuminated by either a 915 nm wavelength diode laser or 525 nm LED and then imaged on the rat’s retina, through a customized lens periscope, to stimulate a photovoltaic prosthetic retinal chip or normal retinal areas, respectively. This system relies on the DMD features, pixel size of a few microns and high power efficiency, to project patterns with high spatial resolution at high light intensities at the desired wavelength.

The head mounted projection system must be suited for behaving rats’ studies, both in terms of physical properties (weight and dimensions) as well as in the optical parameters design. The main physical constraints of the system are low weight and small dimensions to enable its carrying by a behaving rat. Another challenging task is to project high quality images through the relatively poor optics of the rat’s eye.

In this paper we describe in thorough detail the optical characterization of the designed optical system using computer based optical simulations (ZEMAX) which are then validated by empirical data. The system’s performance in both wavelengths was investigated while changing various parameters, such as the pupil size, object size and the tolerance of the system to changes in the object’s position (vertex). Finally, we present electrophysiological data demonstrating the capability of the model to record visual evoked potentials (VEP) in response to visual patterns projected by the head mounted DMD.

## Results

### System design and dimensions

**Figure 1:**
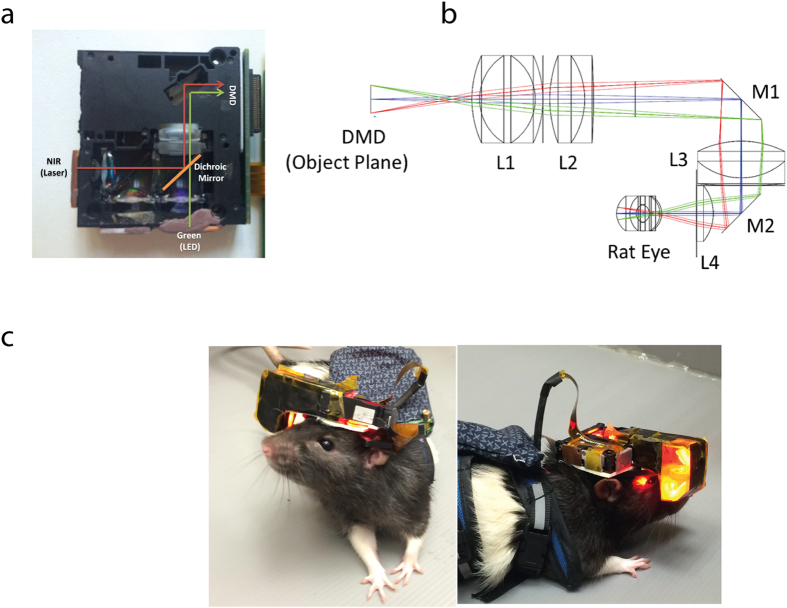
Head mounted DMD system – general and optical design. (**a**) Light sources were customized for both stimulation of the normal vision and photodiode based retinal prosthesis with 525 nm wavelength LED and 915 nm wavelength diode laser, respectively. (**b**) Ray trace analysis of DMD object projected on a rat’s eye model. (**c**) The optical system carried by a behaving rat.

To relay a DMD projected image onto the rat’s retina we developed an optical projection system which takes into account the optical and physical constraints of the rat’s eye (see Methods for details). After following the design criteria and constraints through computer based modeling we reached a design including a lens periscope shown in Fig. 1b which is based on off the shelf three Edmund Optics lenses (lenses 1, 2, 3 in Fig. 1b), two mirrors for the folding (M1,M2), and one off the shelf Linos lens (lens 4 in Fig.1b).

The dimensions of the electronic boards (on the rat’s back Fig. 1c) are 6.5 × 6.5 × 1.5 cm and the head-mounted-projector’s dimensions are 4 × 4 × 1.5 cm. The weight of the head mounted system is 28 gr, which is in line with other reported optical mounted systems^13^. The DMD and its optical path part of the system are fitted to the rat’s head using a customized head plate and adaptor.

### Optical performance of the system: Simulation study

Using Zemax simulation we studied the effect of pupil diameter, object size and vertex distance on the image quality obtained at the retinal plane. As a measure of image quality we employed the Modulation Transfer Function (MTF). The effect of various parameters on the quality of the image at the retinal plane was studied for images with spatial frequency of 1 up to 20 CPM (cycles per mm). When studying the image quality for visible light (wavelength of 525 nm), we addressed the maximal image quality at 17 CPM, which corresponds to the reported visual acuity of about 1 CPD (cycles per degrees) in rats^14^, assuming nodal point distance of 3.39 mm^15^. On the other hand, when studying image quality for prosthetic activation with NIR light (wavelength of 915 nm) we studied retinal image quality at 10 CPM corresponding to the expected resolution achieved with a theoretical implant arrays with electrodes pitch of 50 μm.

### The effect of pupil diameter on MTF

**Figure 2:**
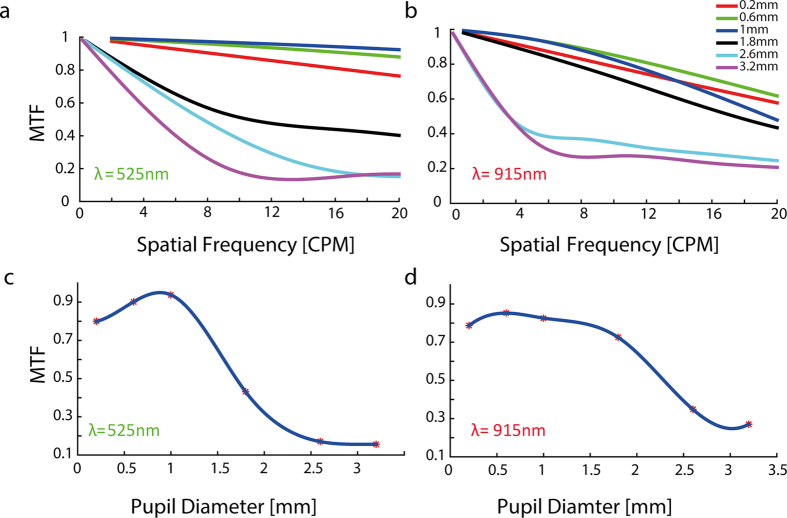
The effect of pupil size on MTF. (**a**) Central sagittal MTF for wavelength of 525 nm (**b**) Central sagittal MTF for wavelength of 915 nm. Each color represents different pupil size. (**c**) The MTF at 17 CPM as a function of pupil diameter for wavelength of 525 nm. (**d**) The MTF at 10 CPM as a function of pupil diameter for wavelength of 915 nm.

Image quality is known to decrease with pupil size in humans^16^ as well as in rodent^17^. To study the effect of pupil diameter on the MTF we ran ZEMAX simulations for pupil diameters ranging from 0.2 mm to 3.2 mm for both visible (525 nm) and NIR (915 nm) light wavelengths. A large pupil diameter was generally associated with decreased image quality in both visible and NIR light, mainly in the high spatial frequencies (Fig. 2a,b). In Fig. 2c the MTF is plotted as a function of pupil diameter at the spatial frequencies of interest: 17 CPM and in Fig. 2d at 10 CPM for wavelengths of 525 nm and 915 nm, respectively. It is worth noting that for the visible wavelength, an optimal MTF of more than 0.9 can be obtained for a pupil diameter of 1 mm, a diameter which will be used for later simulations.

### The effect of field of view size on MTF

**Figure 3:**
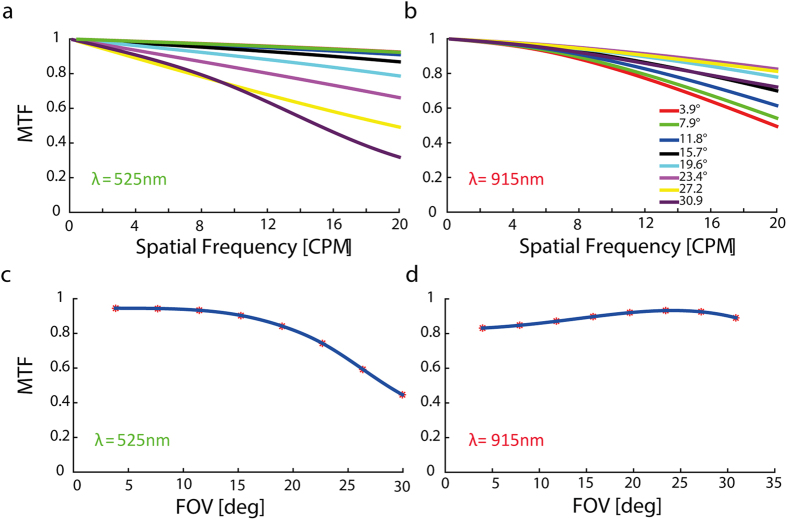
Effect of object size on the MTF. Each color represents a different object height (**a**) Sagittal MTF for various points in the FOV for wavelength of 525 nm (**b**) Sagittal MTF for various points in the FOV for wavelength of 915 nm. (**c**) Sagittal MTF as a function of FOV at 17 CPM for wavelength of 525 nm (**d**) Sagittal MTF as a function of FOV at 10 CPM for wavelength of 915 nm.

In order to investigate the applicability of the system for local retinal treatment, such as a retinal implants, the retinal image of the activation patterns need to be of a few millimeters in its diameter. Larger retinal stimulation area is associated with the perception of larger field of view (FOV) and is generally desired in studying prosthetic vision (1 mm of retinal activation correlates with about 15 degrees field of view in the rat, where the nodal point is 3.39 mm^15^, as compared to about 3 degrees in humans). However, with larger FOV and retinal activation area, the retinal image quality is expected to be reduced, as the rays are diverged from the optical axis, therefore introducing more spherical aberration. To investigate this effect, we studied the effect of the FOV size on the retinal image MTF (Fig. 3). Smaller FOV was generally associated with better MTF values for the visible wavelength (Fig. 3a). Interestingly this was not the case for the NIR light, where the MTF values for the larger FOV sizes, were better than for smaller FOV (Fig. 3b). To further study this effect we studied the effect of FOV on the spatial frequencies of 17 CPM (Fig. 3c) and 10 CPM (Fig. 3d) for visible and NIR stimuli, respectively, which further highlighted our previous observations.

### Tolerance to position of the eye - Vertex distance

**Figure 4:**
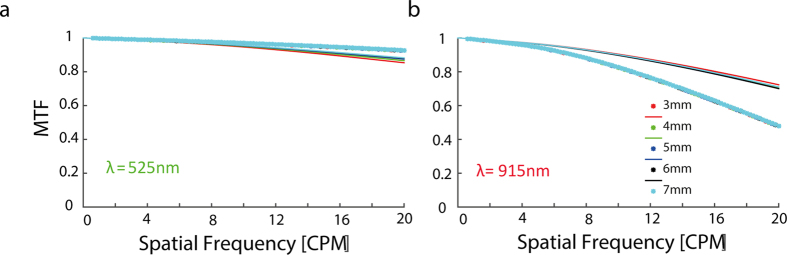
The effect of eye’s position on the MTF. Each color represents a different eye-object distance. The optimal eye-object distance is 5 mm. (**a**) Sagittal MTF as a function of spatial frequency for various vertex distances for wavelength of 525 nm (periphery at ~30deg). (**b**) Sagittal MTF as a function of CPM for various vertex distances for wavelength of 915 nm (periphery at ~30deg).

Finally, in order to investigate the effect of system’s position in relation to the eye on the MTF (i.e. the system’s sensitivity to vertex distance alignment) we performed simulations in which we varied the distance between the optical projector and the retina. Figure 4 illustrates the effect of the distance between the retina and the tip of the periscope on the system’s MTF for the different wavelengths. The sagittal MTF for peripheral field (~30deg) for wavelength of 525 nm at 17 CPM and for the wavelength of 915 nm at 10 CPM was approximately 0.9 for the optimal distance (5 mm). Interestingly, changing the vertex distance by 4 mm had no significant effect on both, the central and the peripheral field (data not shown)

To summarize, ZEMAX simulations, revealed that the system can project patterned images at the desired quality on the rat’s retina, at the desired light wavelengths. Optimal image properties were obtained with small pupil size and smaller object’s size. More importantly, image misalignment did not significantly affect image quality, highlighting the system’s invariance to vertex distance and providing flexibility in system’s placement.

### System validation measurements

**Figure 5:**
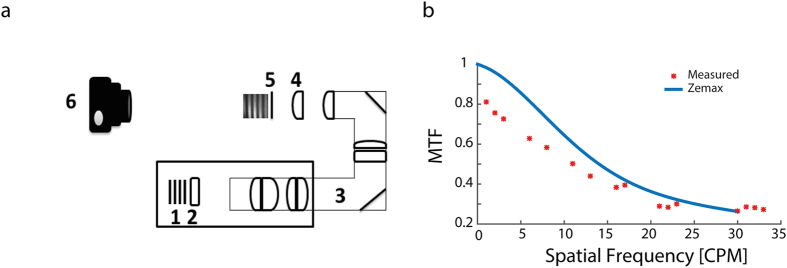
Simulation validation measurements system. (**a**) A schematic diagram of the constructed system for validation of the simulation results; Projected image-object plane (1), DMD system (2), periscope system as previously described (3), a lens modeling the rat’s eye (4), resulting image (on a diffuser (5) ), CCD camera capturing the resulting image (6). (**b**) Obtained versus measured central sagittal MTF as a function of CPM for wavelength of 525 nm (solid line- simulation results, asterisk- measured MTF).

In order to validate the simulation results we constructed a measurement system resembling the actual periscope and animal’s eye system (Fig. 5a), as described in the methods section. We projected images with varying spatial frequencies at 100% contrast and calculated the MTF obtained at an image captured at the image plane according to Michelson contrast equation. Although the calculations do not take into account the MTF degradation resulting from the CCD camera (Imaging Source DMK 21BF04) that was used to acquire the images, a good fit (R^2^ = 0.92) was found between the ZEMAX calculated MTF (Fig. 5b, solid line) and the measured MTF (Fig. 5b, asterix). These data validate the ZEMAX simulations and the feasibility of conducting such simulation studies for characterizing the optical system. As an additional measure of image quality, image distortion was calculated as the mean squared error (MSE) between the captured image and desired image for various CPMs (in which values of MTF >0.5 were obtained). As can be observed the obtained image distortion was low (around 3.5%) and relatively constant across the varying CPMs as expected, (Fig. 3S).

### Visual cortex recording

**Figure 6:**
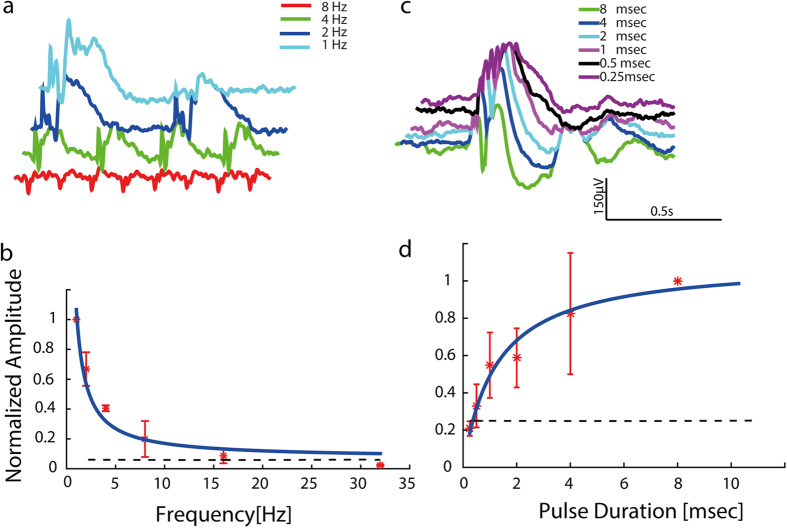
Electrophysiological responses. (**a**) Average VEP response traces for various pulse frequencies for a pulse duration of 4 msec, and averaging of over 200 repetitions. (**b**) The N1-P2 VEP normalized amplitude as a function of repetition rate and power fit R^2^ = 0.97 –solid line. (**c**) Average VEP response traces for various pulse durations at a repetition rate of 1 Hz, and averaging of over 200 repetitions (n = 3). (**d**) The N1-P2 VEP normalized amplitude as a function of pulse duration and polynomial fit –solid line- (n = 3).

Another feature of this novel projection system is the incorporation of visual cortex recording (e.g. visual evoked potentials (VEP) or single unit recordings). This capability is achieved through electrodes implanted into the visual cortex and embedded into the mounting head plate (see *Methods*). We demonstrate this ability through the simultaneous recording of VEPs in response to full field flashes projected through the periscope onto the retina of anesthetized rats. Although the system incorporates two light sources (visible and NIR), as a first step we present responses induced by visible stimuli. To investigate the effect of the stimuli temporal characteristics on the VEP, full field flashes stimuli with varying pulse durations (ranging from 0.25–8 msec) and frequencies (1 Hz-32 Hz) were projected while the VEP was recorded using the AlphaSnR amplification system (Alpha omega ltd). Offline analysis was then performed to calculate the average induced VEP, Fig. 6a depicts a characteristic VEP waveform with the features N1, P2, similar to those previously reported by our group^10^. As expected, the VEP amplitude decreases as a function of temporal frequency (Fig. 6a,b) reaching the noise limit at stimuli frequencies higher than 32 Hz and increases as a function of pulse duration, reaching a plateau at pulses longer than 10 ms can be estimated from the fit graph (Fig. 6c,d). The results suggest that larger animals (>400 gr) are fully capable of carrying this system with no movement constraints while measuring cortical responses to various visual stimuli. Future studies will focus on behavioral vision studies.

The effect of system parameters on the quality of the obtained image, was further investigated by studying the effect of pupil diameter on VEP amplitude (for n = 3 animals). The VEP signals were recorded under three different experimental conditions: non-dilated pupil (1 mm in diameter), inter mediate dilation (on average 2.5 mm in diameter) and fully dilated pupil (5 mm). An alternating grid stimulus with 0.1CPD was projected in all three conditions (Fig. S2.a). As expected the VEP amplitude decreases as the pupil diameter increases (Fig. S2.b) as a result of the degradation in the image quality, in accordance with our simulation studies (Fig. 2c).

## Discussion

In this paper we present a novel head mounted projection system suited for the study of visual performance for both natural and artificial stimuli in behaving rats. Using off the shelf optical and electronic components, and under several physical and optical constraints, we modified a commercially available DMD system to project patterns at 525 nm and 915 nm wavelengths and relayed the projected patterns into close proximity to the rat’s eye through a custom designed and manufactured periscope like lens system. The optimal periscope design was reached using ZEMAX simulations and by optimizing the system’s MTF using the parameters of the readily available optical components. In addition, ZEMAX simulations were used for image quality investigations and to study the effect of various important parameters on image quality. Simulation results revealed that the system has good optical performance with an MTF of higher than 0.9 for the visible wavelength at the desired resolution of 17 CPM for both the central and more peripheral fields (Fig. 3c) and it was higher than 0.8 at 915 nm for the desired resolution of 10 CPM (Fig. 3d). Our investigation of the pupil diameter effect on the obtained MTF illustrated the well-known phenomenon whereby spherical aberrations and wavelength dependent diffraction yield an optimal pupil diameter of 1 and 0.6 mm for wavelengths of 525 nm and 915 nm, respectively (Fig. 2c,d). As was further highlighted by the effect of the pupil diameter on the VEP amplitude (Fig. S2.b).

Similarly, we also noted that using the central field of vision (up to around 20 degrees) will result in better image quality in the desired spatial frequency range (Fig. 3), as expected from the significant effect of spherical aberrations on peripheral image quality. It is worth noting that the system tolerance for displacements in the DMD, to the eye distance is good as can be observed from the system’s invariance to the vertex distance (Fig. 4c,d).

Our simulation results were further validated by measuring the image quality obtained by the projector system’s optics, with good fit between the simulation and measured data.

The low weight of the system (28 gr) and its cortical recordings capabilities will enable the use of this system for a variety of vision research applications. One possible application of the system is the training of freely moving rats to respond to visual stimuli while studying the visual function performance obtained by both natural and artificial vision. The study of visual performance of behaving, in contrast to anesthetized animals can aid in exploring the effect of locomotion on learning enhancement and plasticity^18^. Furthermore, the incorporation of the VEP recording capability into the system further highlights its potential as an investigative tools in freely behaving animals as demonstrated through our preliminary electrophysiological recording obtained during simultaneous pattern projection in a normally sighted animal.

Moreover, this novel approach with its advantageous of high spatial resolution properties, efficient high power patterns projection and wavelength flexibility can aid in evaluation of emerging technologies, such as optoelectronic retinal prosthesis or optogenetic based treatments for blindness. This novel head mounted projection system may prove a vital tool and the first of its kind to the best our knowledge in studying natural and artificial vision in awake and behaving animals, for the evaluation of various treatments or other interventions, such as training.

## Materials and Methods

All animal experiments and procedures were authorized by the Bar Ilan University animal care committee and are in line with the ARVO guidelines for animal experimentations. Long Evans rats (n = 3, weighing 400–500 gr) were used in all electrophysiological experiments.

### Head mounted projection system

The novel head mounted projection system was developed around a DMD projection system engine (Texas Instruments LightCrafter 3000 DLP Platform) at the heart of which is a Digital Mirror Device (display resolution of 684 × 608 and 10.8-μm, micromirror pitch) with its optical projection path, which enables the projection of flexible light patterns with high spatial resolution. Several mechanical and optical adaptations were made, under the constraints of animal anatomy and weight carrying capabilities. Firstly, we separated the DMD and the optical components from the electronic boards, such that only the DMD and the optical components were mounted on the rat’s head while the electronic boards were carried by the rat’s torso. Secondly, the DMD’s R&B original light sources were deactivated while keeping the G source. In addition, a 915 nm laser diode source (500 mW, TEM, China) was added and the two light sources were combined by dichroic mirrors (Fig. 1a) to form a single optical path.

To mount the system’s components on the rat’s head a customized head plate and adaptor were designed and fixated chronically onto the animal’s head by a preparatory surgery, to which the optical system was then connected for the experiments duration (the animal did not chronically carry the system).

### Periscope optical system design suitable for the rat eye

To optimize projection efficiency and obtain a large field of view which rely on the proximity of the projection system to the rat’s eye optical component (i.e. cornea) we developed a periscope like optical system which relays the DMD projected image onto the rat’s retina (Fig. 1b). The choice of the optical components of the periscope-like system, placement and the distance of the system tip from the cornea were based on simulation results obtained using ZEMAX ray tracing software package (see below) while the mechanical design of the device was performed by Solid Edge CAD design tool. The periscope system was built under optical and physical constraints and the final path was optimized using ZEMAX simulations (see later). Firstly, optimal image properties for a projected image resolution of 50 μm for artificially induced vision, which corresponds to 10 CPM for wavelength of 915 nm, is needed. Secondly, a large field of view covering a relatively large area of the retina is desired. Furthermore, optical constrains including the system’s F/#, focal length, the illumination spectrum, the rat’s eye dimensions and the optical parameters according to the Hughes rat eye model^15^ (detailed simulation results of this model may be found in Supplementary Fig. S1a,b)^19^ were extracted. After obtaining the optimal parameters, the mechanical parts were 3D printed by DLP MIIcraftr 3D Printer.

### ZEMAX simulation

We used ZEMAX simulations (ZEMAX Radiant^®^ 2003) to optimize and characterize the optical system performance. Firstly, we investigated the pupil size effect on the system performance as described by the MTF value for both wavelengths (525 nm and 915 nm) for 0–20 CPM and then plotted the MTF value as a function of the radius at spatial frequency of 17 CPM for wavelength of 525 nm (which corresponds to the well-known 1 CPD visual acuity limit in rats). We also plotted the MTF as a function of pupil size for the wavelength of 915 nm at spatial frequency of 10 CPM (corresponding to the required 50 μm image resolution for a theoretical prosthetic retina with pixel pitch of 50 μm). Secondly, we studied the effect the periscope’s distance from the rat’s eye has on the obtained optical performance for both wavelengths for a pupil size of 1 mm. The former simulations were later used for inferring the distance at which the periscope system should be placed from the rat’s eye for optimal system’s performance.

### System validation measurements

To characterize the system’s optical performance and validate the computer based simulations, an optical relay system comprised of the previously described periscope system, a lens (C280TM-B with a focal length of 18.40 mm) and a CCD camera (DMK 21BF04.H, Sony Corp.) were constructed (Fig. 5a). To characterize the system’s MTF we projected patterns with increasing spatial frequency using custom written MATLAB software (The Mathworks, Waltham, MA, USA.) with 100% contrast and calculated the MTF of the resulting CCD captured image (Fig. 5b). The contrast of the captured image was calculated using the Michelson contrast equation which states:


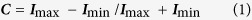


Where I_min_ and I_max_ are the minimum and maximum intensity in the region of interest respectively and C is the image contrast. The MTF was then calculated by dividing the contrast of the captured image by that of the projected image. The calculated MTF was then compared to the MTF obtained from the ZEMAX simulations. For comparison purposes and throughout this work we focused on the sagittal value of the simulated MTF which is the relevant value when patterns perpendicular to the optical axis are considered (at x = 0). As an additional measure of image quality, image distortion was calculated as the mean squared error (MSE) between the captured image and desired image for various CPMs (in which values of MTF >0.5 were obtained). The MSE calculation was performed on the images after edge detection in MATLAB (The Mathworks, Waltham, MA, USA.).

### VEP Recordings

Visual cortex activity induced by visual stimuli projected by the head mounted projector were recorded. Responses to flashes with varying pulse duration (ranging from 0.25 msec 8 msec) and varying frequency (ranging from 1 Hz to 32 Hz) were recorded through cranially implanted screw electrodes which were embedded in the head plate used for mounting the projection and served as electrodes (the implantation procedure was previously described in ref. 10). Signals were acquired (sampled at 1375 Hz, x20 amplification, 4–200 Hz LFP filters) and recorded using AlphaSnR (Alpha omega ltd.). The acquired signals were then analyzed offline (averaged over 200 stimulus repetitions) using custom written MATLAB program (The Mathworks, Waltham, MA, USA).

## Additional Information

**How to cite this article**: Arens-Arad, T. *et al*. Head mounted DMD based projection system for natural and prosthetic visual stimulation in freely moving rats. *Sci. Rep.* **6**, 34873; doi: 10.1038/srep34873 (2016).

## Supplementary Material

Supplementary Information
