# Supplementary material for: Head mounted DMD based projection system for natural and prosthetic visual stimulation in freely moving rats
Source: Sci Rep. 2016 Oct 12;6:34873. doi: 10.1038/srep34873 (PMC5059752; doi:10.1038/srep34873)
Supplement: Supplementary Information [file srep34873-s1.pdf]

Head mounted DMD based projection system for natural and prosthetic visual stimulation in freely moving rats

*Tamar Arens-Arad[a,b], *Nairouz Farah[a,b], Shai Ben-Yaish[c], Alex Zlotnik[c], Zeev Zalevsky[b,c] and Yossi Mandel*[a,b]

[a]Faculty of Life Sciences, Optometry Track, [b]Bar Ilan's Institute for Nanotechnology and Advanced Materials (BINA), [c]Faculty of Engineering, Bar Ilan University, Ramat Gan, Israel

* These authors contributed equally to this work

Supplementary Material

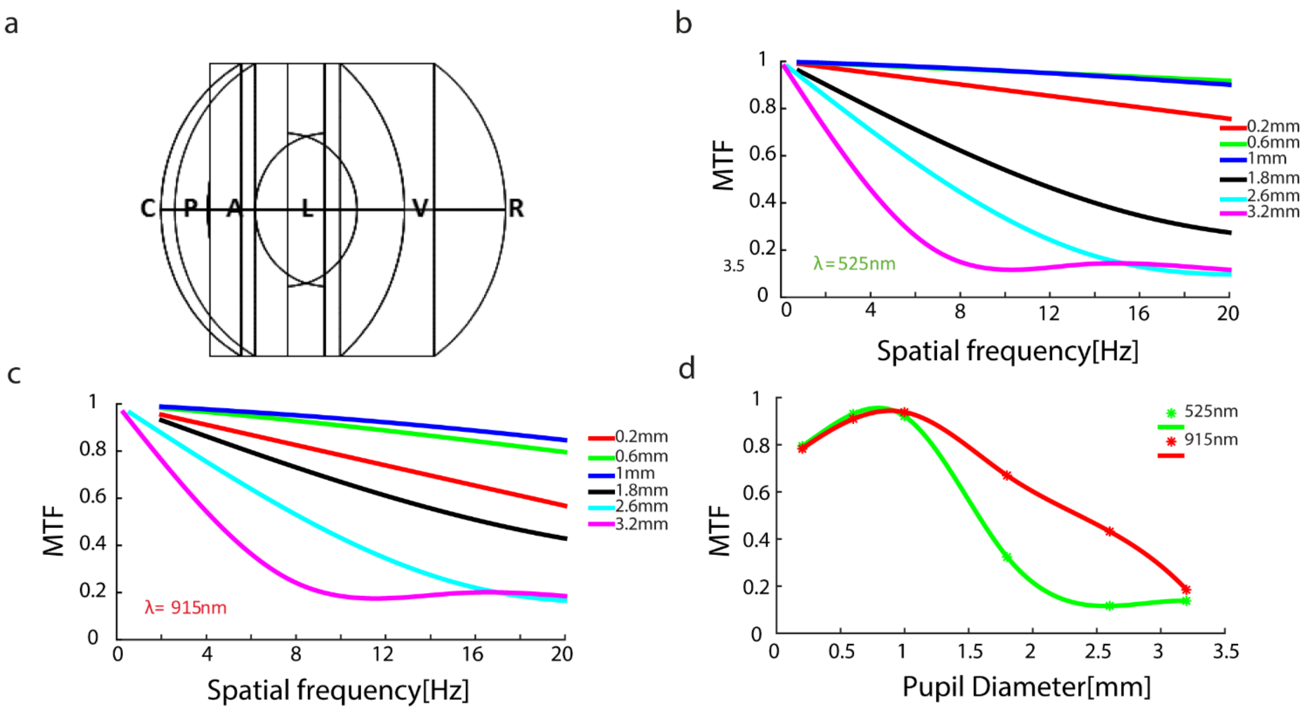

Figure S1: Rat eye model and characteristics: (a) Rat eye model created by ZEMAX software, according to Hughes model. (b) Rat eye central Sagittal MTF as a function of Spatial Frequency for wavelength 525nm. (c) Rat eye central Sagittal MTF as a function of Spatial Frequency for wavelength 915nm. (d) Rat eye central sagittal MTF at 17 CPM and 10 CPM for wavelengths of 525nm and 915nm respectively.

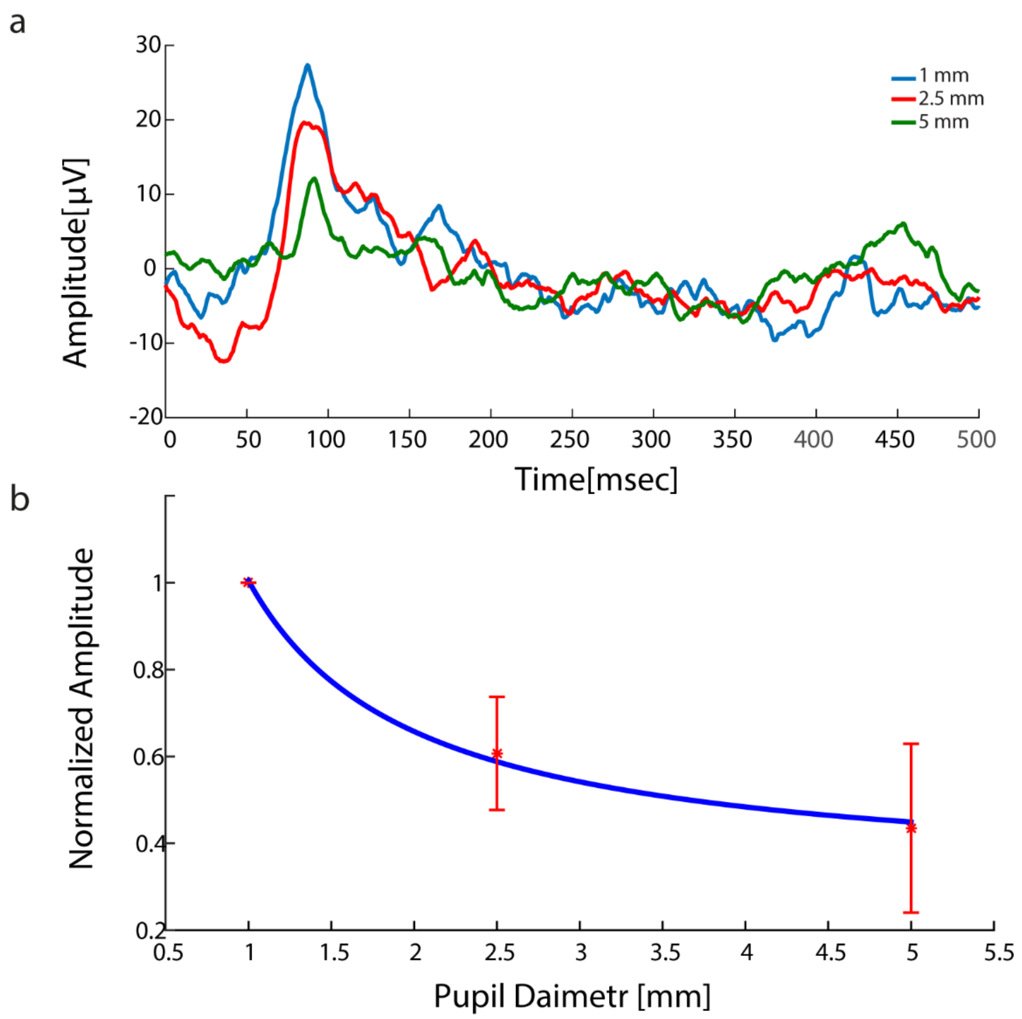

Figure S2: Electrophysiological responses for various pupil sizes. (a) Average VEP response for various pupil diameters, and averaging of over 200 repetitions. (b) The N1-P2 VEP amplitude as a function of pupil diameter and power fit –solid line (n=3).

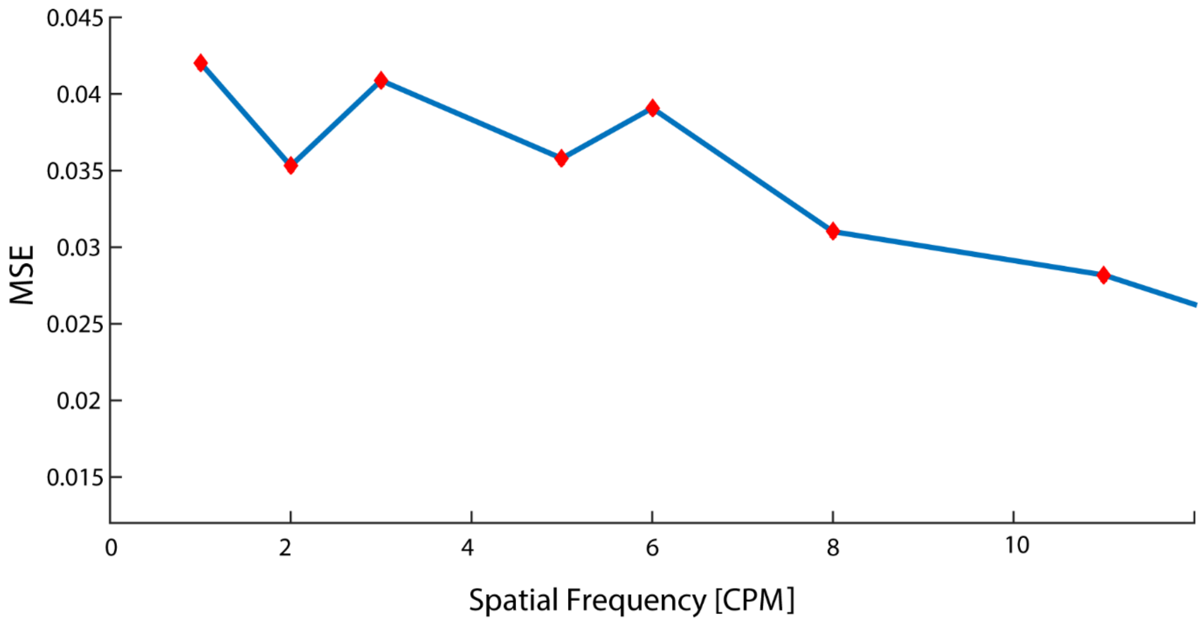

Figure S3: Image distortion as a function of CPM
